# Pro- and Anti-Inflammatory Cytokines in the Context of NK Cell–Trophoblast Interactions

**DOI:** 10.3390/ijms23042387

**Published:** 2022-02-21

**Authors:** Valentina Mikhailova, Polina Grebenkina, Evgeniia Khokhlova, Alina Davydova, Zeina Salloum, Elizaveta Tyshchuk, Valeria Zagainova, Kseniia Markova, Igor Kogan, Sergey Selkov, Dmitry Sokolov

**Affiliations:** 1 Department of Immunology and Intercellular Interactions, Federal State Budgetary Scientific Institution, Research Institute of Obstetrics, Gynecology and Reproductology named after D.O. Ott, 199034 St. Petersburg, Russia; grebenkinap@gmail.com (P.G.); evenka95@gmail.com (E.K.); alyadavydova@gmail.com (A.D.); corbiepost@yandex.ru (Z.S.); tyshhuk.elizaveta@gmail.com (E.T.); kl.markova94@gmail.com (K.M.); selkovsa@mail.ru (S.S.); falcojugger@yandex.ru (D.S.); 2 Department of Immunology, State Budgetary Educational Institution of Higher Professional Education First Pavlov State Medical University of St. Petersburg under the Ministry of Healthcare of the Russian Federation, 197022 St. Petersburg, Russia; 3 Department of Artificial Reproduction Technologies, Federal State Budgetary Scientific Institution, Research Institute of Obstetrics, Gynecology, and Reproductology named after D.O. Ott, 199034 St. Petersburg, Russia; zagaynovav.al.52@mail.ru (V.Z.); ikogan@mail.ru (I.K.)

**Keywords:** NK cells, trophoblast, NK-92, pNK, JEG-3, TNFα, TGFβ, CD56, PBMC

## Abstract

During pregnancy, uterine NK cells interact with trophoblast cells. In addition to contact interactions, uterine NK cells are influenced by cytokines, which are secreted by the cells of the decidua microenvironment. Cytokines can affect the phenotypic characteristics of NK cells and change their functional activity. An imbalance of pro- and anti-inflammatory signals can lead to the development of reproductive pathology. The aim of this study was to assess the effects of cytokines on NK cells in the presence of trophoblast cells in an in vitro model. We used TNFα, IFNγ, TGFβ and IL-10; the NK-92 cell line; and peripheral blood NK cells (pNKs) from healthy, non-pregnant women. For trophoblast cells, the JEG-3 cell line was used. In the monoculture of NK-92 cells, TNFα caused a decrease in CD56 expression. In the coculture of NK cells with JEG-3 cells, TNFα increased the expression of NKG2C and NKG2A by NK-92 cells. Under the influence of TGFβ, the expression of CD56 increased and the expression of NKp30 decreased in the monoculture. After the preliminary cultivation of NK-92 cells in the presence of TGFβ, their cytotoxicity increased. In the case of adding TGFβ to the PBMC culture, as well as coculturing PBMCs and JEG-3 cells, the expression of CD56 and NKp44 by pNK cells was reduced. The differences in the effects of TGFβ in the model using NK-92 cells and pNK cells may be associated with the possible influence of monocytes or other lymphoid cells from the mononuclear fraction.

## 1. Introduction

Natural killer cells (NK cells) are cytotoxic, innate immunity lymphocytes that are characterized by the presence of the surface receptors CD45 and CD56, as well as the absence of the linear differentiation receptors CD3, CD14 and CD19 [1]. Mature NK cells predominantly circulate in the peripheral blood (pNK). Resident populations of NK cells are found in lymphoid organs (spleen, tonsils, lymph nodes, Peyer’s patches of the intestine and thymus) and non-lymphoid organs (liver, lungs and uterus) [2,3]. One example of tissue-resident cells is uterine NK cells. During pregnancy, NK cells accumulate in the decidual membrane, forming a pool of decidual NK cells (dNKs) [4]. Fewer NK cells reside in the endometrium in non-pregnant women [4]. 

NK cells express various receptors that regulate cytotoxicity towards trophoblast cells [5,6]. The receptors have not yet been clearly defined; however, on the basis of their expression, it is possible to separate NK cells into cytotoxic or regulatory populations [7]. The cells of the uteroplacental complex form a cytokine microenvironment that can influence the phenotype and functions of NK cells and regulate their interactions with trophoblast cells.

Extravillous trophoblast cells produce TNFα and its receptors [8], as well as TGFβ [9]. Under the influence of a conditioned medium from trophoblast cells, peripheral blood monocytes differentiate into macrophages that are similar in their characteristics to the macrophages of the decidua and produce TGFβ [10]. Through the secretion of TGFβ, decidual macrophages can suppress the cytotoxic activity of decidual NK cells [11]. TGFβ stimulates the acquisition of pNK features that are characteristic of dNK cells [12] and also affects the functions of NK cells [13]. In addition, TGFβ reduces IFNγ secretion by NK cells, blocks signals from the NKG2D and CD244 receptors of NK cells and contributes to a decrease in the expression of NKp30. As a result, the cytotoxic activity of NK cells ultimately decreases [14,15].

First-trimester decidua macrophages secrete IL-10, TNFα and IFNγ [16,17,18,19]. The IL-10 receptor consists of two subunits and is expressed on many cells, including NK cells [19]. IL-10 inhibits the cytotoxic and secretory functions of NK cells [20]. The increased production of IFNγ and TNFα by macrophages leads to the increased apoptosis of trophoblast cells [16]. It has been shown in mice that IFNγ stimulates a multidirectional change in inhibitory receptor expression in pNK cells [21]. 

In addition to microenvironment cell cytokines, NK cells can directly synthesize cytokines. It has been shown that CD56+ pNK cells produce IFNγ, TNFα and TGFβ in the first trimester of pregnancy [22]. Some populations of dNK cells synthesize TNFα [12,23,24], IFNγ [12,14], TGFβ [24] and IL-10 [23,24] during the first trimester of physiological pregnancy. An imbalance of pro- and anti-inflammatory cytokines in the uteroplacental complex can lead to the development of reproductive pathology. For example, IL-10 gene expression in the placenta is reduced in preeclampsia [25], while IFNγ secretion is increased [26], when compared to normal pregnancy. The increased production of the cytokines IFNγ and TNFα by dNK cells and placental macrophages is associated with the development of miscarriage [16,23]. Thus, a wide range of cytokines have been described in the uteroplacental complex, yet there are insufficient data on the effects of cytokines on the phenotype and functional state of NK cells during contact interaction with trophoblast cells. Therefore, the aim of this work was to assess the effects of the cytokines TNFα, IFNγ, TGFβ and IL-10 on the receptor profile and cytotoxicity of NK cells in coculture with JEG-3 trophoblast cells.

## 2. Results

### 2.1. Influence of Cytokines on the Phenotype of the NK-92 Cell Line in Monoculture and under Co-Cultivation Conditions with the JEG-3 Cell Line

We used a model system of contact cultivation with trophoblast cells, which we described previously [27]. The phenotype of NK-92 cells was assessed after their cultivation without (hereinafter referred to as monoculture) and in the presence of trophoblast cells (hereinafter referred to as coculture). 

#### 2.1.1. Effect of TNFα on the Phenotype of the NK-92 Cell Line in Monoculture and under Co-Cultivation Conditions with the JEG-3 Cell Line

**Figure 1:**
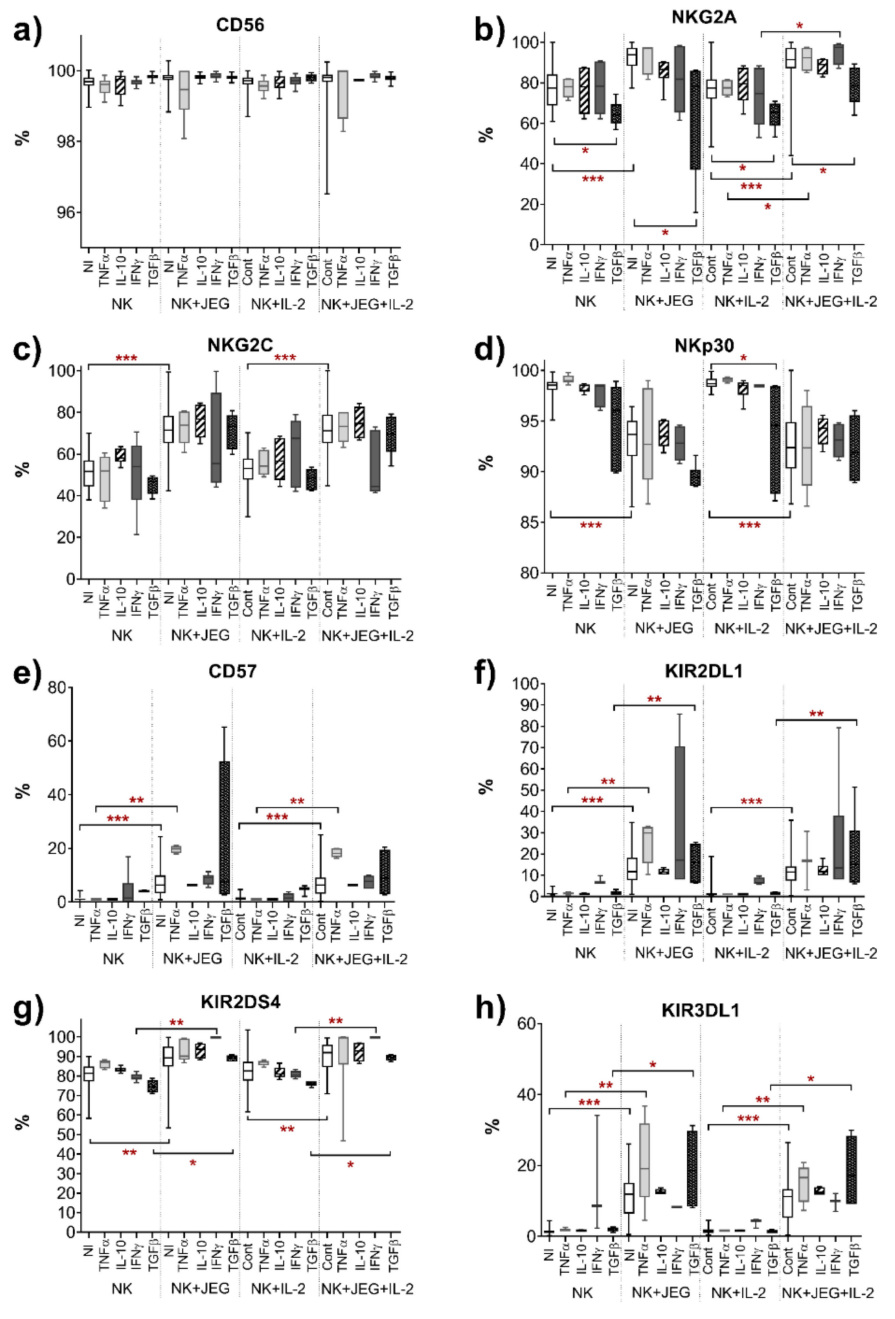
The number of NK-92 cells with phenotypes (**a**) CD56+, (**b**) NKG2A+, (**c**) NKG2C+, (**d**) NKp30+, (**e**) CD57+, (**f**) KIR2DL1+, (**g**) KIR2DS4+ and (**h**) KIR3DL1+ after culturing in monoculture and coculture with JEG-3 cell line, with cytokines TNFα, IL-10, IFNγ and TGFβ. NI (no inducer)—cultured without inducers. Cont (control)—cultured with IL-2. The experiments with each of the cytokines were conducted separately, twice, with three replicates for each experiment. Significant differences: *—*p* < 0.05, **—*p* < 0.01 and ***—*p* < 0.001 (Kruskal–Wallis test).

**Figure 2:**
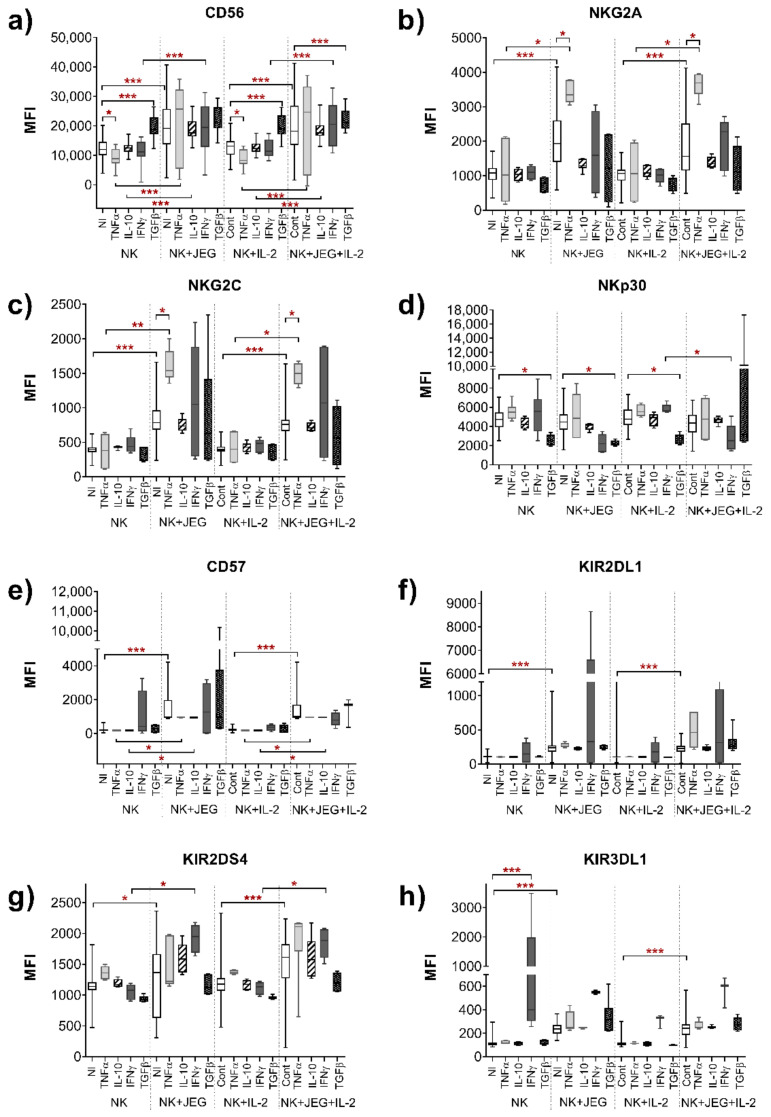
The intensity of (**a**) CD56, (**b**) NKG2A, (**c**) NKG2C, (**d**) NKp30, (**e**) CD57, (**f**) KIR2DL1, (**g**) KIR2DS4 and (**h**) KIR3DL1 receptor expression by NK-92 cells after culturing in monoculture and coculture with JEG-3 cell line, with cytokines TNFα, IL-10, IFNγ and TGFβ. NI (no inducer)—cultured without inducers. Cont (control)—cultured with IL-2. The experiments with each of the cytokines were conducted separately, twice, with three replicates for each experiment. Significant differences: *—*p* < 0.05, **—*p* < 0.01 and ***—*p* < 0.001 (Kruskal–Wallis test).

The number of NK-92 cells did not change after the addition of TNFα to the monoculture (Figure 1). In the monoculture, the addition of TNFα resulted in a decrease in the expression of the CD56 receptor by NK-92 cells compared to cultivation without TNFα in both IL-2+ and IL-2-free media (Figure 2a).

In the coculture, compared with the monoculture, the number of NK-92 cells with the phenotype CD57+, KIR2DL1+, KIR3DL1+ and CD127+ increased in the presence of TNFα in the culture medium without IL-2 (Figure 1e,f,h and Figure S1h). In the case of culturing in a medium with IL-2 and TNFα, the number of NKG2A+, CD57+, KIR3DL1+ and CD127+ NK-92 cells increased in the coculture compared to the monoculture (Figure 1b,e,h and Figure S1h).

In the case of the coculture, the addition of TNFα led to an increase in the expression of NKG2A and NKG2C receptors compared to the cell coculture without TNFα. These changes were revealed after cultivation in media either without IL-2 or with IL-2 (Figure 2b,c). 

In the presence of TNFα, the expression of the receptors CD56, NKG2A, NKG2C, CD57 and CD122 by NK-92 cells increased in the coculture compared to the monoculture in media either without IL-2 or with IL-2 (Figure 2a–c,e and Figure S2g). 

#### 2.1.2. Effect of IL-10 on the Phenotype of the NK-92 Cell Line in Monoculture and under Co-Cultivation Conditions with the JEG-3 Cell Line

In the case of using medium without IL-2, the addition of IL-10 to the medium caused an increase in the number of KIR2DL3+ and CD161+ cells for the NK-92 cell line in the coculture compared to monoculture (Figure S1c,f). When cultivated in a medium with IL-2 and IL-10, the number of KIR2DL3+ cells of the NK-92 line also increased in the coculture compared to monoculture (Figure S1c).

The expression of the CD56 and CD57 receptors by NK-92 cells increased in the coculture compared to monoculture when using IL-10-containing medium either with IL-2 or without it (Figure 2a,e).

#### 2.1.3. Influence of IFNγ on the Phenotype of the NK-92 Cell Line in Monoculture and under Co-Cultivation Conditions with the JEG-3 Cell Line

IFNγ caused an increase in the expression of KIR3DL1 by NK cells only in the case of the monoculture of NK-92 cells in a medium without IL-2 (Figure 2h). 

In IFNγ-containing media without IL-2 or with IL-2, the number of NK-92 cells with the KIR2DS4+ and CD161+ phenotype increased in the coculture compared to the monoculture (Figure 1g and Figure S1f). In the medium with IL-2 and IFNγ, we observed that the number of NK-92 cells with the NKG2A+ and CD215+ phenotype also increased in the case of the coculture compared to the monoculture (Figure 1b and Figure S1i).

In the medium without IL-2 but with IFNγ, the expression of the NKp44 receptor by NK-92 cells increased in the case of the coculture compared to the monoculture (Figure S2a). In the presence of IFNγ, the expression of CD56, KIR2DS4 and CD127 by NK cells increased in the coculture compared to the monoculture, when using the medium either without IL-2 or with IL-2 (Figure 2a,g and Figure S2h). In the medium with IL-2 and IFNγ, the expression of the NKp30 receptor by NK-92 cells was reduced in the case of the coculture compared to monoculture (Figure 2d).

#### 2.1.4. Effect of TGFβ on the Phenotype of the NK-92 Cell Line in Monoculture and under Co-Cultivation Conditions with the JEG-3 Cell Line

TGFβ in monoculture reduced the relative number of NK-92 cells with the NKG2A+ KIR2DL4+ phenotype compared to cultivation without TGFβ, in the case of culturing NK cells in media both without IL-2 and with IL-2 (Figure 1b and Figure S1b). TGFβ in combination with IL-2 in monoculture led to a decrease in the number of NK-92 cells with the NKp30+ and CD117+ phenotype compared to the monoculture cultured only with IL-2 (Figure 1d and Figure S1e). TGFβ in the monoculture increased the intensity of CD56 expression by NK-92 cells and reduced the intensity of NKp30 expression in the media both without IL-2 and with IL-2 (Figure 2a,d). 

The expression of the receptors KIR2DL4, CD161 and CD127 by NK-92 cells increased in the coculture compared to the monoculture in the presence of TGFβ in the culture media both without IL-2 and with IL-2 (Figure S2b,f,h).

The addition of TGFβ to the coculture resulted in a decrease in the number of NK-92 cells expressing NKG2A and CD62L compared to the coculture without TGFβ (Figure 1b and Figure S1d). These changes were established using culture media both without IL-2 and with IL-2. 

The number of NK-92 cells with the KIR2DL1+ KIR2DS4+ KIR3DL1+ KIR2DL3+ phenotype increased in the coculture compared to monoculture in medium supplemented with TGFβ (Figure 1a–h and Figure S1c). These changes were established using culture media both without IL-2 and with IL-2. 

TGFβ in medium without IL-2 reduced the intensity of NKp30 expression by NK-92 cells in coculture compared with the coculture without TGFβ (Figure 2d). In the medium with IL-2, TGFβ increased the expression of the CD56 receptor by NK-92 cells in coculture compared to the coculture without TGFβ (Figure 2a). The expression of the CD161 receptor by NK cells increased in the coculture after the addition of TGFβ, compared to the coculture without TGFβ, using media both without IL-2 and with IL-2 (Figure S2f). 

**Scheme 1:**
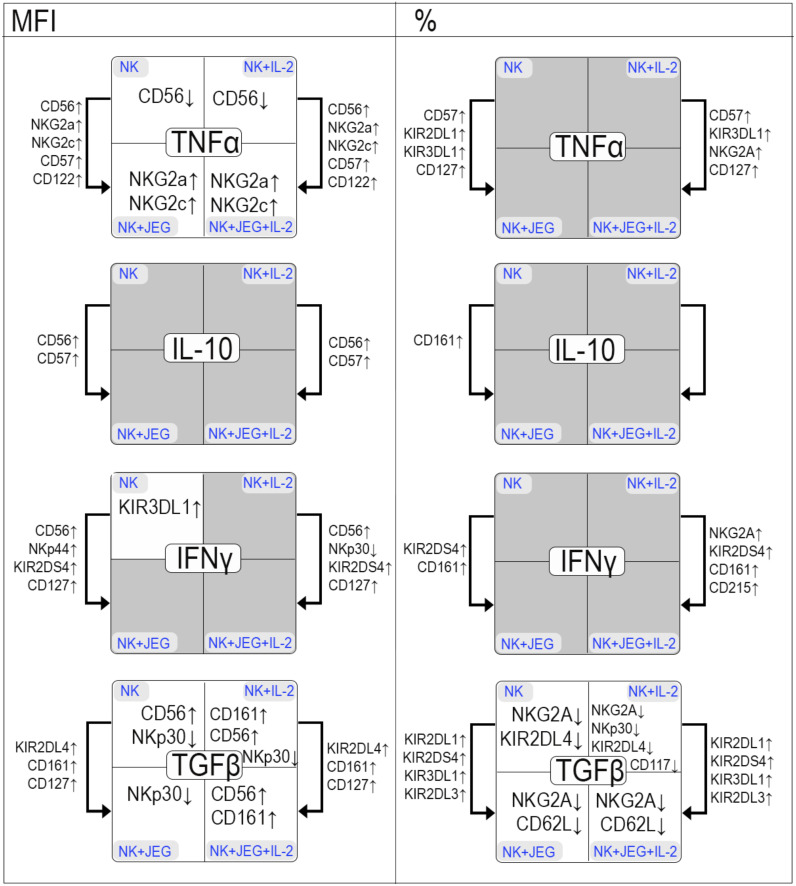
Effect of TNFα, IL-10, IFNγ and TGFβ on NK-92 cells in different culturing variants. The left part of the diagram shows data for the intensity of NK cell expression of receptors (MFI); the right part of the diagram shows data for the number of NK cells. Four cultivation situations are shown in each rectangle: NK cells in monoculture (NK), NK cells cultured with IL-2 (NK+IL-2), NK cells in coculture with JEG-3 trophoblast cells (NK+JEG) and NK cells in coculture with JEG-3 trophoblast cells cultured with IL-2 (NK+JEG+IL-2). Changes in the expression or relative number of NK cells as a result of the addition of a cytokine in each culture situation are shown inside the box. Changes in the expression or number of NK cells in the case of cultivation of cells in coculture compared with monoculture, in the presence of a cytokine in the culture medium, are shown by arrows to the left and right of the rectangles.

The results of the assessment of the effects of the cytokines TNFα, IL-10, IFNγ and TGFβ are shown in Scheme 1.

### 2.2. Cytotoxicity of NK-92 Cell Line after Culture with Cytokines

**Figure 3:**
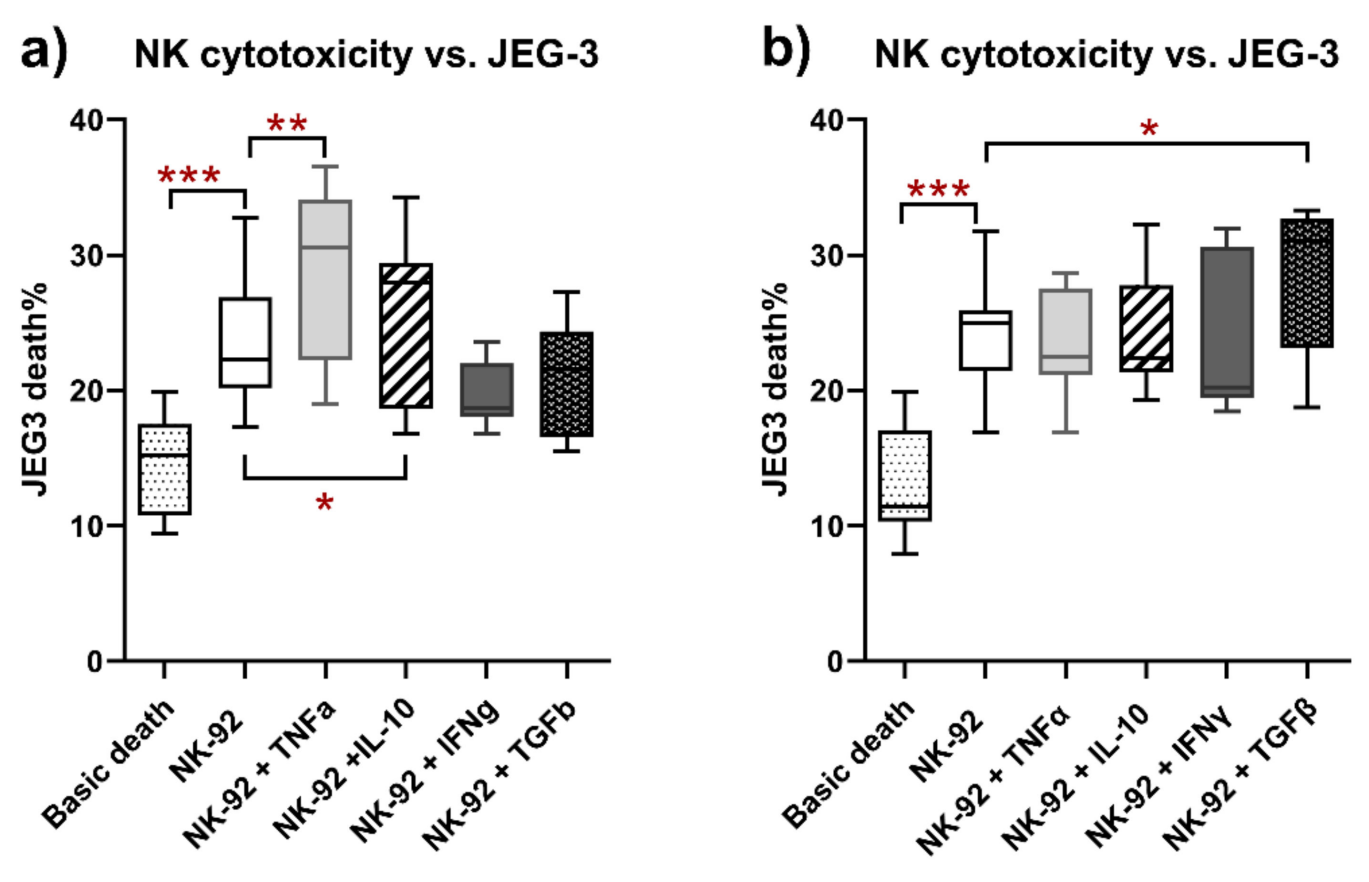
The NK-92 cells’ cytotoxicity towards JEG-3 cells after 24 h (**a**) and 96 h (**b**) of NK cell pre-culturing with cytokines TNFα, IL-10, IFNγ and TGFβ. Experiments with each cytokine were conducted separately, three times, using four replicates in each experiment. Significant differences: *—*p* < 0.05, **—*p* < 0.01 and ***—*p* < 0.001 (Kruskal–Wallis test).

The death of the target cells increased when NK cells were added to JEG-3 trophoblast cells compared to the baseline death levels. After the pre-cultivation of NK cells for 24 h in the presence of TNFα and IL-10, an increased cytotoxicity of NK cells was found compared to the function of inactivated NK cells (Figure 3a). After the pre-culture of NK cells for 96 h in the presence of TGFβ, the cytotoxicity of the NK cells was increased compared to the function of unstimulated NK cells (Figure 3b).

### 2.3. Phenotype of Peripheral Blood NK Cells in Monoculture and under Co-Cultivation Conditions with the JEG-3 Cell Line

**Figure 4:**
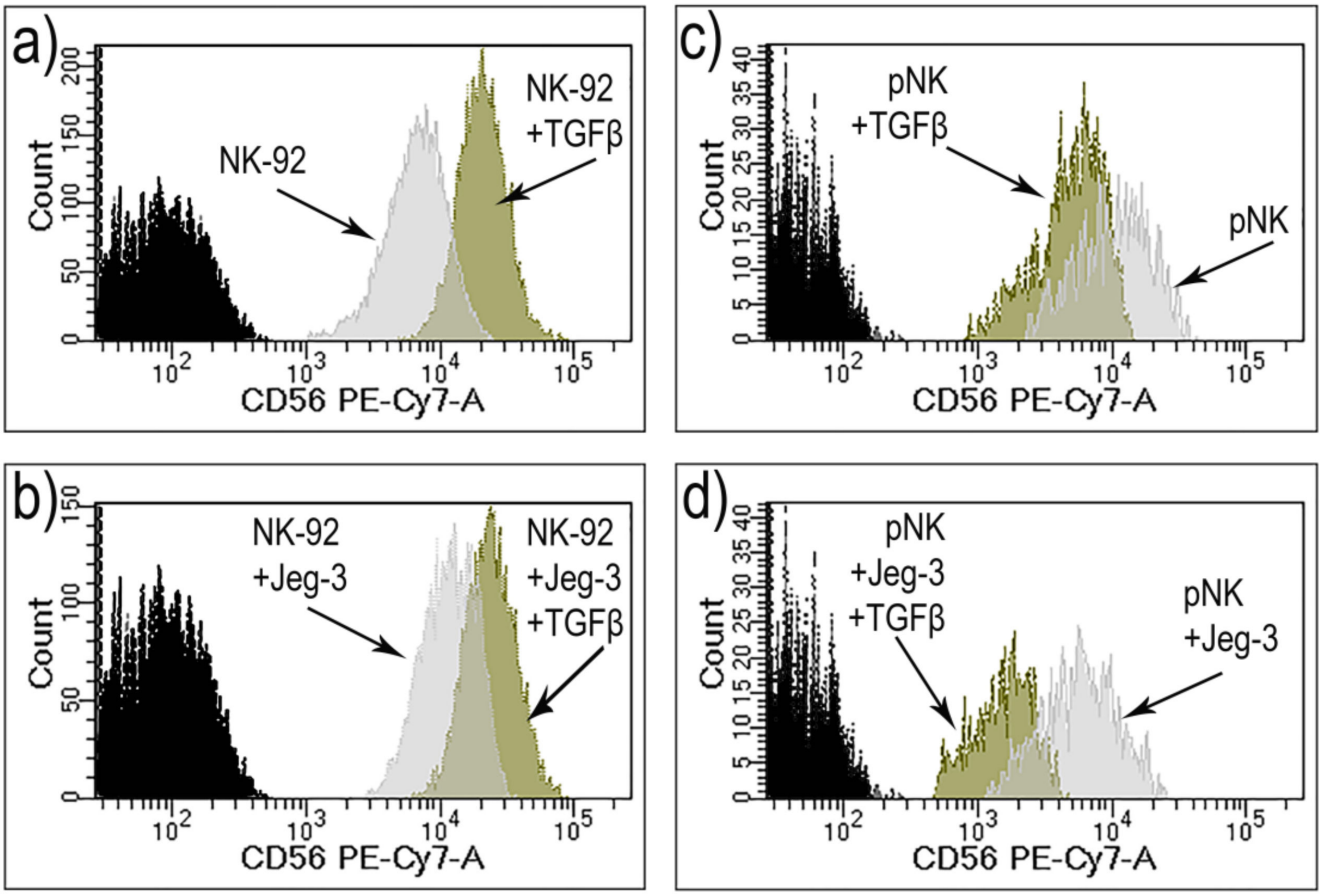
The intensity of CD56 receptor expression by NK-92 cells (**a**,**b**) and peripheral blood NK cells (**c**,**d**). Cells were cultured without inducers (grey) and with TGFβ (dark green). Isotype control is shown in black.

The analysis of the phenotype of pNK cells before cultivation revealed no differences between two subgroups of healthy, non-pregnant women. In this regard, all the examined women were combined into one group to assess the effect of the JEG-3 trophoblast cell line on pNK cells. Since both the phenotypic profile of NK-92 cells and their cytotoxicity changed after a 96-h cultivation in the presence of TGFβ, this cytokine was chosen to analyze phenotypic changes in a system using peripheral blood NK cells. Figure 4 shows representative graphs for CD56 expression by NK-92 cells and pNK cells.

**Figure 5:**
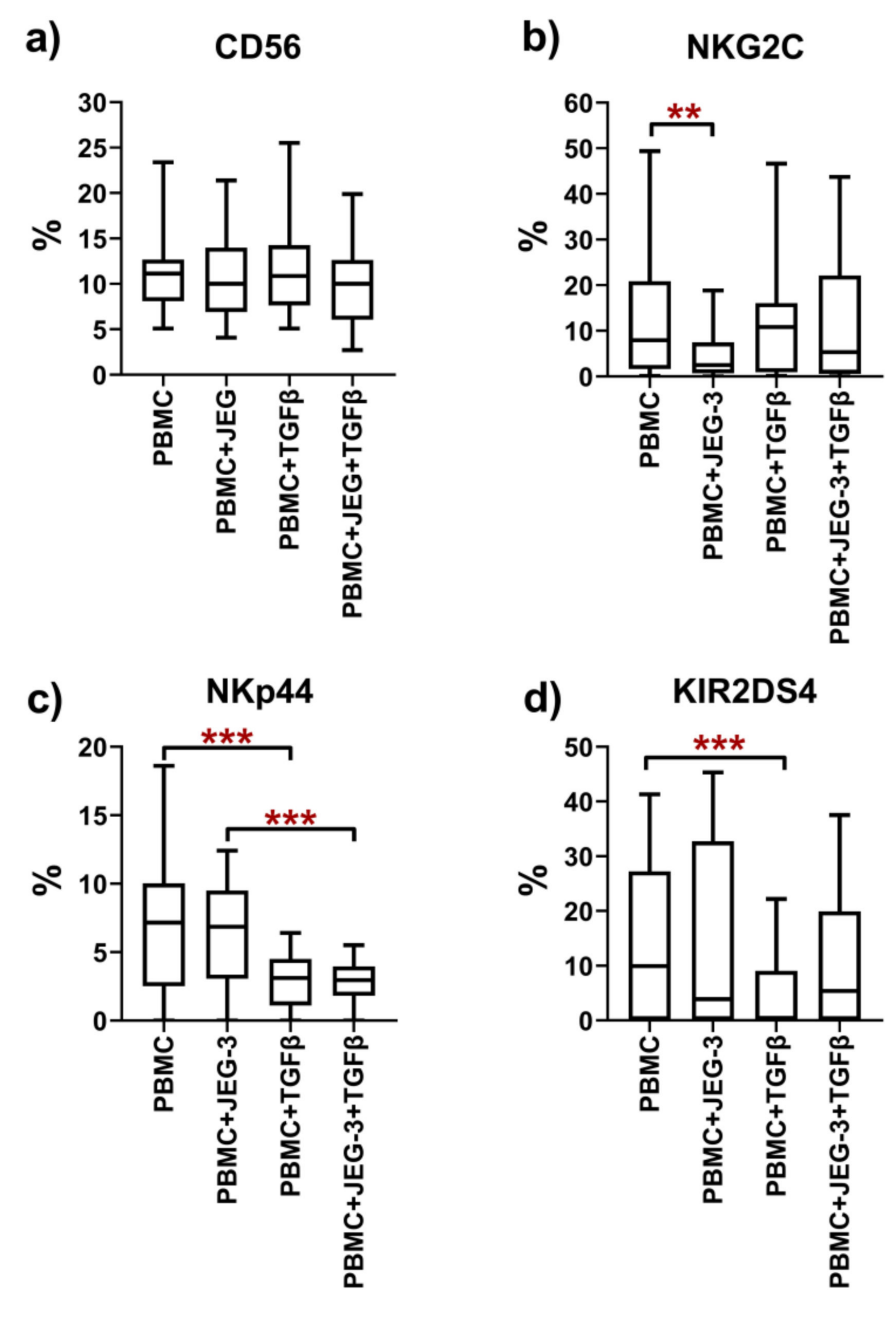
The number of pNK cells with (**a**) CD56+, (**b**) NKG2C+, (**c**) NKp44+ and (**d**) KIR2DS4+ phenotypes after monoculture without inducers (peripheral blood mononuclear cells (PBMC)) and with TGFβ (PBMC + TGFβ), and in coculture with JEG-3 cells without cytokines (PBMC + JEG-3) and with TGFβ (PBMC + JEG-3 + TGFβ). PBMCs were collected from healthy, non-pregnant women (*n* = 21). Significant differences: **—*p* < 0.01 and ***—*p* < 0.001 (Wilcoxon matched-pairs rank test).

**Figure 6:**
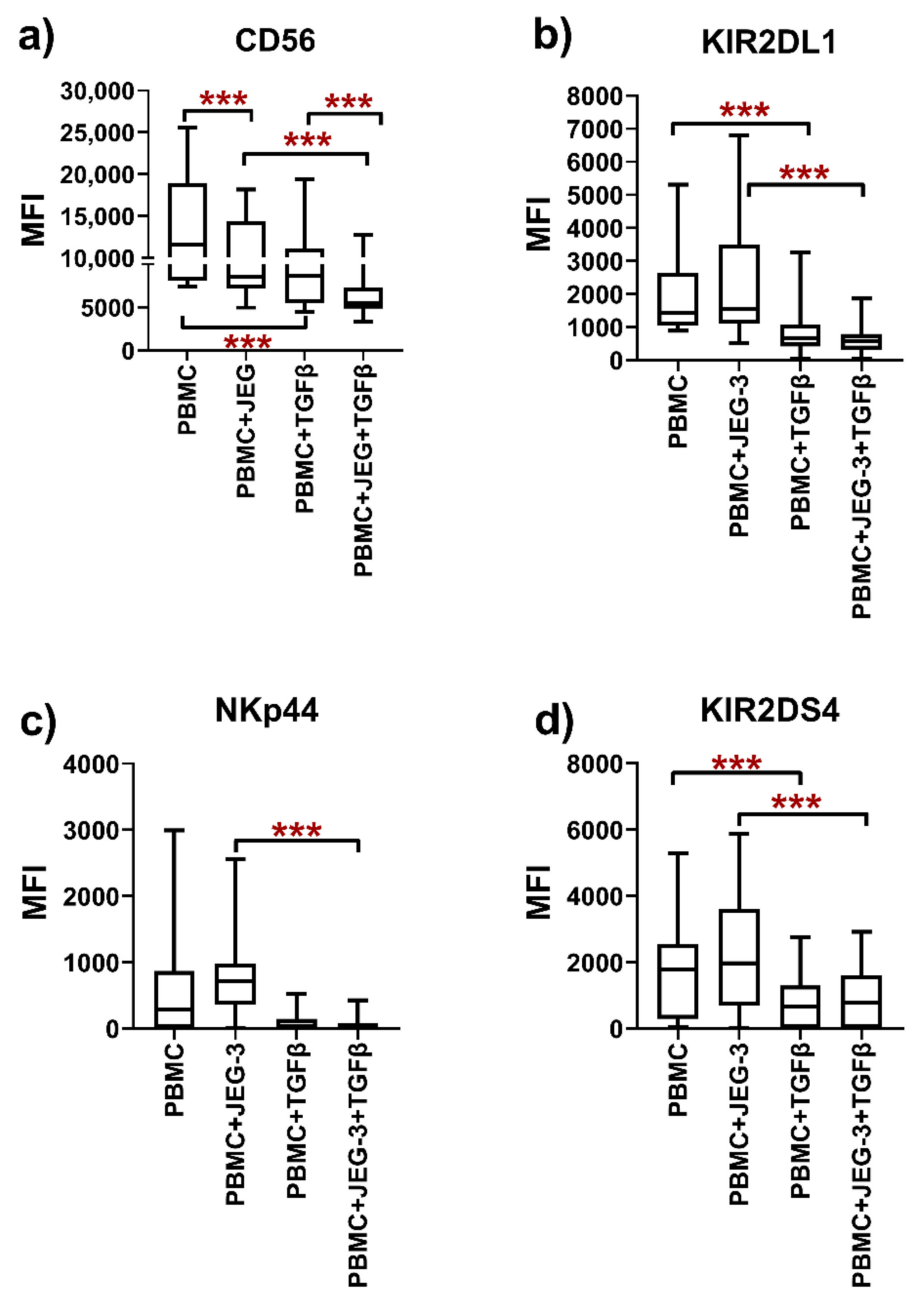
The intensity of (**a**) CD56, (**b**) KIR2DL1, (**c**) NKp44 and (**d**) KIR2DS4 receptor expression by pNK cells after monoculture without inducers (PBMC) and with TGFβ (PBMC + TGFβ), and in coculture with JEG-3 cells without cytokines (PBMC + JEG-3) and with TGFβ (PBMC + JEG-3 + TGFβ). PBMCs were collected from healthy, non-pregnant women (*n* = 21). Significant differences: ***—*p* < 0.001 (Wilcoxon matched-pairs rank test).

After the cultivation of peripheral blood mononuclear cells (PBMC) with TGFβ, the number of pNK cells with the phenotype NKp44+ and KIR2DS4+ (Figure 5c,d) and the expression of CD56, KIR2DL1 and KIR2DS4 (Figure 6) were reduced compared to unstimulated pNK cells.

After culturing with JEG-3 trophoblast cells, the number of pNK cells with the NKG2C+ phenotype and the expression of CD56 by pNK cells were reduced compared to the cells cultivated without trophoblasts (Figure 5a). 

After culturing mononuclear cells in the presence of trophoblast cells and TGFβ, the number of NKp44+ pNK cells (Figure 5) and the expression of the CD56, KIR2DL1, NKp44 and KIR2DS4 receptors by NK cells were reduced compared to pNK cells in coculture with JEG-3 trophoblast cells without the addition of TGFβ (Figure 6).

The intensity of CD56 expression by pNK cells was reduced in the case of the cultivation of mononuclear cells in a medium containing TGFβ with JEG-3 trophoblast cells, compared with a monoculture of PBMCs in a medium with TGFβ (Figure 6).

## 3. Discussion

TNFα is a pleiotropic cytokine [28]. TNFα enhances the activating effect of IL-2 and also stimulates the cytotoxicity of NK cells against target cells in vitro [29,30]. We showed that cultivation with TNFα reduced the expression of CD56 by NK-92 cells. We also found that, after pre-cultivation with TNFα for 24 h, the cytotoxicity of NK-92 cells against JEG-3 trophoblast cells was enhanced. These changes in cell phenotype and function can be regarded as the induction of the CD56dim cytotoxic phenotype in the presence of TNFα.

It has been reported that NK cells, after in vitro cultivation in the presence of feeder cells expressing membrane-bound IL-21, can change their phenotype and begin to express NKG2C and CD57 receptors, retaining their ability to induce cytotoxicity [31]. These changes, together with the acquisition of HLA-DR, are regarded as being characteristic of adaptive NK cells [31]. In women with previous pregnancies, “pregnancy-trained” decidual NK cells have been described, which are characterized by the increased expression of NKG2C [32]. Endometrial NK cells have been shown to express more NKG2A than pNK cells [33]. In the present work, in the case of culture with JEG-3 trophoblast cells, the addition of TNFα caused the increased expression of the activating receptor NKG2C and the inhibitory receptor NKG2A. An increased number of CD57+ NK-92 cells and increased expression of CD57 were also found in the case of coculture compared to monoculture. These changes in the NK-92 cell line phenotype after exposure to trophoblast cells may reflect the regulatory effect of trophoblast cells and the induction of a memory-like NK cell phenotype in the case of pregnancy. TNFα is produced by endometrial cells in the secretory phase of the menstrual cycle [34], as well as by dNK cells in the first trimester of pregnancy [23]. In this regard, the effect of TNFα in combination with the contact interaction with trophoblast cells cannot be regarded solely as pro-inflammatory.

At the same time, it is known that the secretion of TNFα, as well as IFNγ, by dNK cells is increased in spontaneous miscarriage [23]. However, we found that the cytotoxicity of NK cells against JEG-3 cells after the preliminary cultivation of NK-92 cells for 96 h in the presence of TNFα did not change compared to that for non-activated NK cells. It is likely that the absence of changes in the cytotoxicity of NK cells against JEG-3 cells after cultivation with TNFα is associated with a short-term effect of the activation of NK-92 cells and the loss of the activated state after a 96-h incubation.

Another pro-inflammatory NK cell cytokine is IFNγ. We found that IFNγ caused an increase in the expression of the activation receptor KIR3DL1 by NK-92 cells in monoculture. After cultivation in a medium with IFNγ, the effect of trophoblast cells on NK-92 cells was preserved: an increase in the expression of CD56 and KIR2DS4 compared to that in the monoculture was detected. Song X. et al. described a correlation between the cytotoxic function of NK-92 cells and their secretion of IFNγ upon interaction with K562 target cells [35]. Despite the fact that IFNγ is a pro-inflammatory cytokine, we were unable to detect differences between the cytotoxicity of NK cells cultured with IFNγ and the activity of unstimulated NK cells against JEG-3 cells. This lack of effect could be associated with both the concentration of IFNγ used and the time of cell stimulation. Previously, we found that, in the case of the short-term cultivation of NK-92 cells and JEG-3 cells, IFNγ at a similar concentration (1000 IU/mL) caused an increase in the cytotoxic activity of NK cells compared to that of non-activated cells [36]. It has been shown that, in the case of the long-term cultivation (72 h) of JEG-3 cells in the presence of IFNγ, the expression of HLA-G by trophoblast cells is increased [37]. Thus, according to our data and previously published data, the interaction of NK cells and trophoblast cells in the presence of IFNγ does not merely involve contact cytolysis, but also includes changes in the phenotypic characteristics of both NK cells and trophoblast cells, which can also affect their cytotoxic function.

IL-10 is an immunoregulatory cytokine whose production has been demonstrated in dNK cells [38] and decidual macrophages [18]. IL-10 was found to affect the interaction of dNK cells and the dendritic cells of the uteroplacental complex in a mouse model [39]. We did not establish a direct effect of IL-10 in either the mono- or coculture of the NK-92 and JEG-3 cell lines. With IL-10 in the culture medium, the expression of CD56 and CD57 in NK cells increased in the coculture compared to the monoculture. These changes in phenotype indicate the persistent modulating effect of trophoblast cells in the presence of IL-10.

In the current study, we found that after 24 h of preculturing, IL-10 strongly improved the cytotoxic activity of NK-92 cells towards JEG-3 cells. It was previously established that, when IL-10 was added to a model system to assess contact cytolysis by NK cells towards trophoblast cells, it caused an increase in the cytotoxicity of NK-92 cells [36]. After preculturing with IL-10, pNK cells have been shown to possess increased lytic activity against K562 cells [40,41] and an increase in granzyme B content [41]. In addition, in the case of cultivation for 24 h, IL-10 has been shown to reduce the expression of HLA-G by JEG-3 cells [37], which could explain the increased cytotoxicity of NK-92 cells to JEG-3 cells that we observed. Thus, the results obtained are consistent with the data in the literature. 

TGFβ, involved in the regulation of many physiological and pathological processes, is one of the most powerful immunosuppressive cytokines [42]. The trophoblast JEG-3 cell line produces TGFβ [43,44]. Co E.C. et al. showed that decidual macrophages, by secreting TGFβ, can reduce the cytotoxicity of NK-92 cells and dNK cells towards K562 cells [11]. We found that under the influence of TGFβ, NK cells increased the expression of CD56 and also reduced the expression of cytotoxic NKp30 receptors. TGFβ is reported to suppress the expression of the CD16 receptor by CD56bright NK cells, stimulating the differentiation of CD56bright CD16-NK cells [45]. It has also been shown that TGFβ inhibits the cytotoxicity of NK cells against dendritic cells by reducing the expression of NKp30 by NK cells [15]. Thus, the changes in CD56 and NKp30 that we established can be regarded as the induction of their regulatory phenotype.

At the same time, according to our data, TGFβ causes a decrease in the number of NK-92 cells with the NKG2A+ and KIR2DL4+ phenotype in monoculture. Hawke L.G. et al. showed that after pNK cells were cultured with IL-15 and TGFβ, they acquired an ILC1-like phenotype and an altered expression of the transcription factors Eomes and T-bet [46]. In response to TGFβ in the culture system with IL-15, the expression of both activating and inhibitory receptors, including NKG2A, by ILC1-like cells increased [46]. Thus, our data complement the earlier results described in the literature. 

In coculture with the JEG-3 cell line, TGFβ also caused an increase in CD56 expression by NK-92 cells, apposing their phenotype to the regulatory one. In the coculture system, TGFβ also affected trophoblast cells. It has been shown that TGFβ can stimulate the invasion of JEG-3 trophoblast cells [47]. The inhibition of signaling from TGFβ in JEG-3 cells leads to a decrease in the proliferation of trophoblast cells [48] and their ability to migrate and invade [43,49]. Trophoblast JEG-3 cells express the immunoregulatory glycan-binding protein galectin-1 (Gal1) [50]. Gal1’s ligand is T cell Ig and mucin domain-containing protein 3 (Tim-3), expressed by dNK cells [12]. TGFβ induces the upregulation of Tim-3 expression by dNK cells [12]. The Tim-3/Galecin-9 interaction inhibits the cytotoxicity of NK cells against HTR-8 trophoblast cells [12]. However, we found that after the preliminary cultivation of NK cells in the presence of TGFβ for 24 h, the death of trophoblast cells did not differ from that for the control. 

In a study using mice, TGFβ suppressed the NK cell-specific transcription factor Eomes in spleen NK cells and induced them to acquire an ILC1-like phenotype [51]. Using ILCs derived from the salivary glands of mice, it was demonstrated that ILC1-like cells contain more granzyme B and C mRNA than NK cells. In knockout mice not expressing TGFβR2, granzyme C expression was reduced in ILC1-like cells. [51]. In our work, we detected an increase in the death of JEG-3 cells only after 96 h of the preliminary incubation of NK cells with TGFβ. It is possible that our results demonstrating the increased cytotoxicity of NK cells after long-term exposure to TGFβ are associated with the transdifferentiation of NK cells into ILC1 cells with higher cytotoxicity. 

In order to confirm the immunomodulatory effect of TGFβ on NK cells in coculture with trophoblast cells, we assessed the phenotype of pNK cells in our model system. Previously, we found that the cytotoxicity of pNK cells against JEG-3 trophoblast cells decreased during the menstrual cycle, and did not differ in the secretory phase of the cycle from that in the first trimester of pregnancy [44]. In this regard, to assess the effect of trophoblast cells on the pNK phenotype, we used PBMCs from healthy women, obtained in the second phase of the menstrual cycle. We found that in the presence of the JEG-3 trophoblast cell line, the expression of CD56 by pNK cells and the relative number of NKG2C+ pNK cells were reduced. According to data in the literature, macrophages suppress the cytotoxic function of NK cells through the secretion of TGFβ [11,52,53]. PBMCs contain monocytes, which, under cultivation, can differentiate into macrophages. Trophoblast cells can enhance the secretion of cytokines by monocytes/macrophages. Awoyemi T. et al. showed that syncytiotrophoblast microvesicles obtained from healthy pregnant women induced the secretion of a spectrum of cytokines, including TNFα, IL-8, IL-6, VEGF, IL-1β and GM-CSF, by monocyte-like cells of the THP-1 line [54]. The combined inhibitory effect of monocytes/macrophages and trophoblast cells explains the observed decrease in the expression of CD56 and the activation receptor NKG2C. 

We also detected the reduced expression of CD56 and the activation receptor NKp44, and a reduced number of pNK cells with the NKp44+ phenotype, when TGFβ was added to a PBMC monoculture as well as to a coculture of PBMCs with JEG-3 cells. However, in our model with NK-92 cells, CD56 expression increased in the monoculture after cultivation with TGFβ. These differences may also be associated with the influence of monocytes on the phenotype of NK cells in PBMCs. This assumption is consistent with the previously established differences in the cytotoxicity of NK cells in PBMCs and NK cells isolated from PBMC fractions [55]. It should be noted that a decrease in the expression of receptors by pNK cells in the presence of TGFβ affected not only the activation receptors but also an inhibitory receptor—KIR2DL1. The observed decrease in KIR2DL1 expression may have been associated with a general suppression of the expression of many NK cell receptors by trophoblast cells in the presence of monocytes. 

We found that in the presence of TGFβ in the culture medium, pNK cells had a lower expression of CD56 in coculture compared with monoculture. TGFβ has been reported to inhibit the production of progesterone and estradiol by JEG-3 cells [56]. It has also been shown in vitro that as a result of exposure to pregnancy hormones, including progesterone, estradiol and prolactin, THP-1 cells can change their secretory profile and begin to produce cytokines characteristic of M2 macrophages [57]. It is possible that the inhibitory effect of trophoblast cells and monocytes in the experiments with PBMCs was enhanced by exogenous TGFβ.

## 4. Materials and Methods

### 4.1. Cell Cultures

We used the NK-92 cell line, which reproduces the main features of natural killer cells (NK cells), in addition to the K562 myelogenous leukemia cell line and JEG-3 trophoblast cells (ATCC, Gaithersburg, MD, USA); all cells were cultured in line with the manufacturer’s recommendations. All the cell culture experiments were carried out in a humid atmosphere at 37 °C under 5% CO_2_.

### 4.2. Peripheral Blood Mononuclear Cells

This study included 21 non-pregnant women with regular menses and an uncomplicated obstetric-gynecological and somatic history. The age range of the group was 29.1 ± 6 years (M ± SD). The group was divided into subgroups depending on obstetric histories: healthy, non-pregnant women (subgroup 1, *n* = 8) and healthy, fertile, non-pregnant women with a history of one or more pregnancies that ended in term delivery (subgroup 2, *n* = 13). Peripheral blood was obtained by venipuncture after an overnight fast, in the secretory phase of the menstrual cycle, after the ultrasound monitoring of ovulation. Blood sampling was carried out in the secretory phase, since we previously showed that NK cells could change their functional activity in relation to trophoblast cells during the menstrual cycle and in the secretory phase, and did not differ in cytotoxicity from the NK cells of pregnant women in the first trimester [58]. The exclusion criteria for both subgroups were exacerbations of chronic diseases; the manifestation of acute inflammatory disease, including antiphospholipid syndrome; external genital endometriosis stage 3–4; anomalies in the development of the genital organs; obesity grade 2–3; a hereditary form of high-risk thrombophilia; diabetes mellitus types 1 and 2; hormone therapy (particularly combined oral contraceptives); or refusal to participate in the study program.

### 4.3. Cytokines

The cytokines IL-2 (‘Roncoleukine’, BioTech, Saint Petersburg, Russia), TNFα (50 IU/mL, R&D Systems, Minneapolis, MN, USA, cat. 210-TA), IFNγ (1000 IU/mL, R&D Systems, Minneapolis, MN, USA, cat. 285-IF), TGFβ (5 ng/mL, R&D Systems, Minneapolis, MN, USA, cat. 240-B) and IL-10 (10 IU/mL, R&D Systems, Minneapolis, MN, USA, cat. 217-IL) were utilized as inducers.

### 4.4. Assessment of the Phenotype of the NK-92 Cell Line after Cultivation with JEG-3 Trophoblast Cells and Cytokines

We used the method described previously in [27]. Before the experiment, JEG-3 cells were introduced into the wells of a flat-bottomed 24-well plate (200,000 cells/mL) in complete DMEM growth medium (Biolot, St. Petersburg, Russia, cat.1.3.5.). After 24 h, the cells formed a confluent monolayer. Then, NK-92 cells were added to the wells with JEG-3 cells (200,000 cells/mL). IL-2 (500 IU/mL) and one of the cytokines TNFα (50 IU/mL), IL-10 (10 IU/mL), IFNγ (1000 IU/mL) or TGFβ (5 ng/mL) were added to the wells. The cells were then cultured for 96 h, after which they were centrifuged (200× *g*, 22 °C, 10 min) and treated with an Fc receptor-blocking reagent (MACS, Teterow, Germany, cat. 130–059-901). Next, the cells were treated with fluorescently-labeled monoclonal antibodies against the phenotypic receptors CD45 (cat. 347464) and CD56 (cat. 557747); cytokine receptors CD127 (cat. 560549), CD122 (cat. 557323) and CD215 (cat. FAB1471N); differentiation receptors CD117 (cat. 333233), CD161 (cat. 556080), CD57 (cat. 333169) and CD62L (cat. 559772); and cytotoxicity receptors CD94/NKG2A (cat. 555889), NKG2C (cat. 748168), NKG2D (cat. 562498), NKp44 (cat. 558564), NKp30 (cat. 558407), KIR2DL3 (cat. FAB2014A), KIR2DL4 (cat. FAB2238P), KIR3DL1 (cat. FAB12251G), KIR2DL1 (cat. FAB1844F) and KIR2DS4 (cat. 564375) (R&D Systems, Minneapolis, MN, USA; Becton Dickinson, Franklin Lakes, NJ, USA). Isotype antibodies (R&D Systems, Minneapolis, MN, USA; Becton Dickinson, Franklin Lakes, NJ, USA) were used as a control for nonspecific binding. Receptor expression was assessed on a FACSCanto II flow cytometer (Becton Dickinson, Franklin Lakes, NJ, USA).

### 4.5. Evaluation of the Cytotoxicity of the NK-92 Cell Line after Pre-Cultivation with Cytokines

NK-92 cells were pre-cultured in a 24-well plate in the presence of TNFα (50 IU/mL), IL-10 (10 IU/mL), IFNγ (1000 IU/mL) or TGFβ (5 ng/mL) for 24 h or 96 h. The incubation with the cytokines was carried out for 24 h, since it was previously established that the incubation of NK-92 cells with the supernatants of placenta samples led to a change in the expression of surface cell receptors [59]. The incubation time of 96 h was chosen to match the conditions of the experiments for the co-cultivation of NK-92 cells and JEG-3 cells. The cells were then centrifuged (200× *g*, 22 °C, 10 min) and added to the target cells at a ratio of 5:1 (effector:target), and the cytotoxic function of NK cells was assessed according to the intensity of target cell death. JEG-3 trophoblast cells were used as targets, as described previously [36].

### 4.6. Evaluation of the Phenotype of pNK Cells after Cultivation with JEG-3 Trophoblast Cells and Cytokines

First, 24 h before the experiment, JEG-3 cells were placed in a 96-well flat-bottom plate, with 20,000 cells per 100 µL of complete DMEM growth medium (BioloT, St. Petersburg, Russia, cat.1.3.5.). One day later, peripheral blood mononuclear cells (PBMCs) were isolated from peripheral blood using the standard method of centrifugation in a density gradient solution of Ficoll (ρ = 1.077, BioloT, St. Petersburg, Russia, cat.1.2.8.1.). Then, the medium was removed from the wells with a trophoblast cell monolayer and PBMCs were placed into the wells at 100,000 cells per 100 μL of complete growth medium (DMEM). To maintain the viability of the PBMCs, we added IL-2 (200 IU/mL) to all the wells, while TGFβ (5 ng/mL) was added to some of the wells, and the cells were incubated for 96 h. The plate was then centrifuged for 5 min at 200× *g*, at 22 °C. The conditioned medium was removed from the wells, and 100 µL of Versene solution (BioloT, St. Petersburg, Russia, cat. 1.2.3.2.) was added. After 3 min, the cells were resuspended, transferred to a round-bottom plate, and then centrifuged at 200× *g*, at 22 °C, for 5 min. The supernatant was removed, and a reagent for blocking Fc receptors (MACS, Teterow, Germany, cat. 130–059-901) was added to the cells. Then, the cells were treated with monoclonal antibodies against the CD3 (cat. 560176), CD56 (cat. 557747), CD45 (cat. 347464), CD14 (cat. 565283), KIR2DS4 (cat. 564375), CD122 (cat. 557323), CD127 (cat. 560549), NKp44 (cat. 558564), NKG2D (cat. 562498) (Becton Dickinson, Franklin Lakes, NJ, USA), KIR3DL1 (cat. FAB12251G), KIR2DL4 (cat. FAB2238P), KIR2DL3 (cat. FAB2014A), KIR2DL1 (cat. FAB1844F), NKG2C (cat. FAB138P) and CD215 (cat. FAB1471N) (R&D Systems, Minneapolis, MN, USA) receptors according to the manufacturer’s instructions. PNK cells were analyzed in PBMCs as lymphocytes with the CD45+ CD14-CD3- CD56+ phenotype using the previously published gating strategy [27]. To assess cell death, some cells were treated with 7-amino-actinomycin D (7-AAD) (cat. 420403, BioLegend, San Diego, CA, USA). The death of NK cells after cultivation in mono- and coculture was no more than 2%. Isotype antibodies (Becton Dickinson, Franklin Lakes, NJ, USA and R&D Systems, Minneapolis, MN, USA) were used as controls. Receptor expression was assessed using a FACS Canto II flow cytometer (Becton Dickinson, Franklin Lakes, NJ, USA).

### 4.7. Statistical Data Analysis

The data were statistically processed using the GraphPad Prism 8 program. We used the Shapiro–Wilk test to assess the normality of the data distributions. Using the Bartlett test, we estimated the homogeneity of variances. Due to the variances being unequal, we further used non-parametric statistics for data analysis (Mann–Whitney U test and Kruskal–Wallis test).

### 4.8. Ethical Approval

The research was conducted in line with the Code of Ethics of the World Medical Association (Helsinki Declaration). The local ethical committee of the Research Institute of Obstetrics, Gynecology, and Reproductology named after D.O. Ott approved the research (protocol No. 107). 

## 5. Conclusions

Here, we analyzed the effect of TNFα, IFNγ, TGFβ and IL-10 on the receptor profile and cytotoxic activity of NK cells. The pro-inflammatory cytokines IFNγ and TNFα stimulated the cytotoxic phenotype of NK cells. We showed that IFNγ promoted an increase in the expression of the activation receptor KIR3DL1 by NK-92 cells. TNFα caused a decrease in CD56 expression by NK-92 cells and stimulated their cytotoxic function. The anti-inflammatory cytokine IL-10 did not affect the expression of receptors by NK-92 cells. In the case of co-cultivation with trophoblast cells, the effect of the cytokines on NK cells was different. TNFα increased the expression of both the activating NKG2C receptor and the inhibitory NKG2A receptor by NK-92 cells. In this regard, TNFα in combination with contact interaction with trophoblast cells has a regulatory effect on NK cells and contributes to the induction of a phenotype similar to that of memory cells. TGFβ, in the case of monoculture and coculture with trophoblast cells, stimulated NK-92 cells to acquire a regulatory phenotype. When TGFβ was added to a monoculture of PBMCs, as well as to a coculture of PBMCs and JEG-3 cells, a reduced expression of CD56 and the activation receptor NKp44 by pNK cells was revealed. Differences in the effect of TGFβ in the model using NK-92 cells and pNK cells may be associated with the possible influence of monocytes from the mononuclear fraction. Further studies are required to establish the full effect of trophoblast cells on the expression of activating and inhibitory NK cell receptors using primary dNK cells and/or decidual macrophages.

## Data Availability

The data presented in this study are available on request from the corresponding author.

## Supplementary Materials

The following supporting information can be downloaded at: https://www.mdpi.com/article/10.3390/ijms23042387/s1.

## Abbreviations

7-AAD—7-amino-actinomycin D; dNK—decidual NK cell; ILCs—innate lymphoid cells; NK cell—natural killer cell; pNK—peripheral blood NK cell; PBMCs—peripheral blood mononuclear cells.
