# Supplementary material for: Pro- and Anti-Inflammatory Cytokines in the Context of NK Cell–Trophoblast Interactions
Source: Int J Mol Sci. 2022 Feb 21;23(4):2387. doi: 10.3390/ijms23042387 (PMC8878424; doi:10.3390/ijms23042387)
Supplement: Supplementary file 1 [file ijms-23-02387-s001.zip › ijms-1593258-supplementary.pdf]

International Journal of *Molecular Sciences*

MDPI

Pro- and Anti-Inflammatory Cytokines in the Context of NK Cell–Trophoblast Interactions

Valentina Mikhailova, Polina Grebenkina, Evgeniia Khokhlova, Alina Davydova, Zeina Salloum, Elizaveta Tyshchuk, Valeria Zagainova, Kseniia Markova, Igor Kogan, Sergey Selkov, Dmitry Sokolov

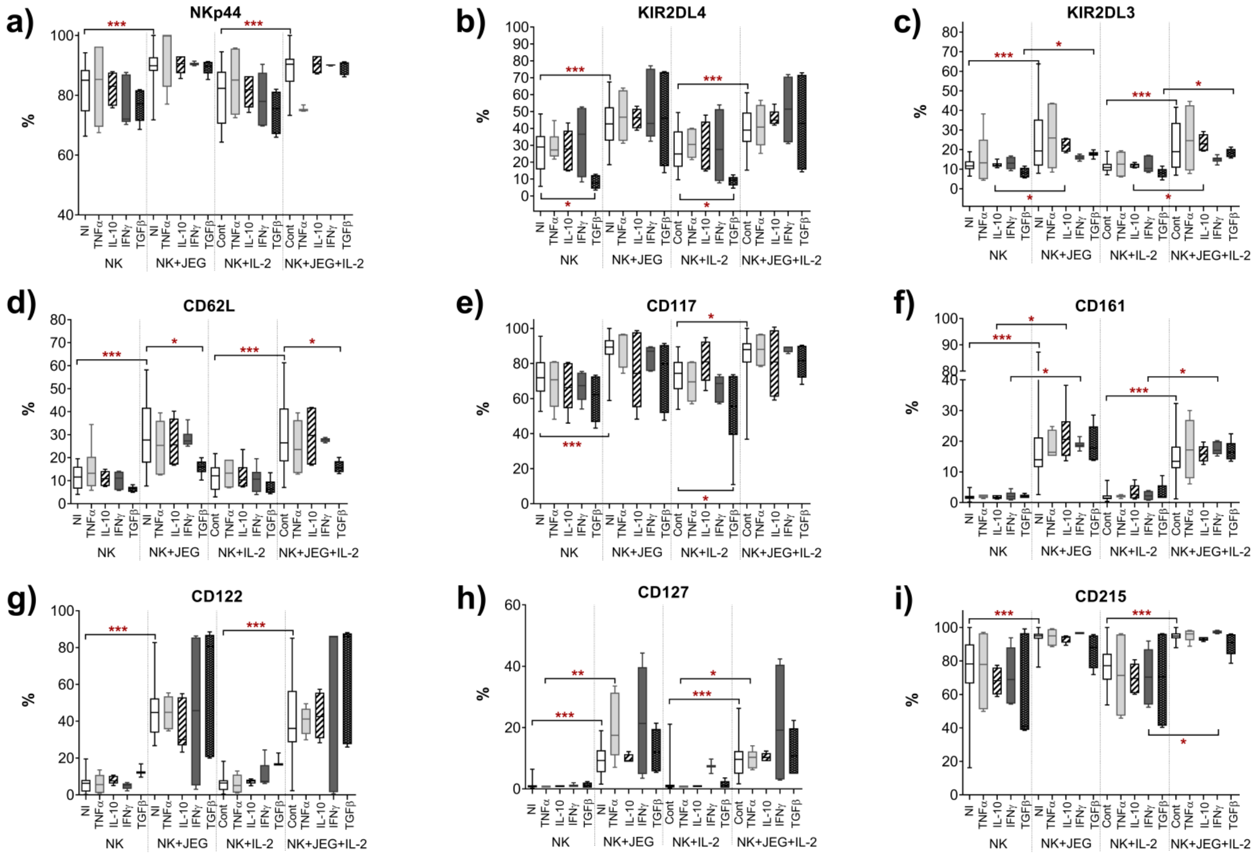

Supplementary Figure 1. The number of NK-92-line cells with **(a)** NKp44+, **(b)** KIR2DL4+, **(c)** KIR2DL3+, **(d)** CD62L+, **(e)** CD117+, **(f)** CD161+, **(g)** CD122+, **(h)** CD127+ and **(i)** CD215+ phenotypes, after culturing in monoculture and coculture with JEG-3 cells, with cytokines TNFα, IL-10, IFNγ and TGFβ. NI (no inducer)—culturing without inducers. Cont (control)—culturing with IL-2. The experiments with each of the cytokines were conducted separately, twice, using three replicates in each experiment. Significant differences: *—p<0.05, **—p <0.01 and ***—p <0.001 (Kruskal–Wallis test).

International Journal of
Molecular Sciences

MDPI

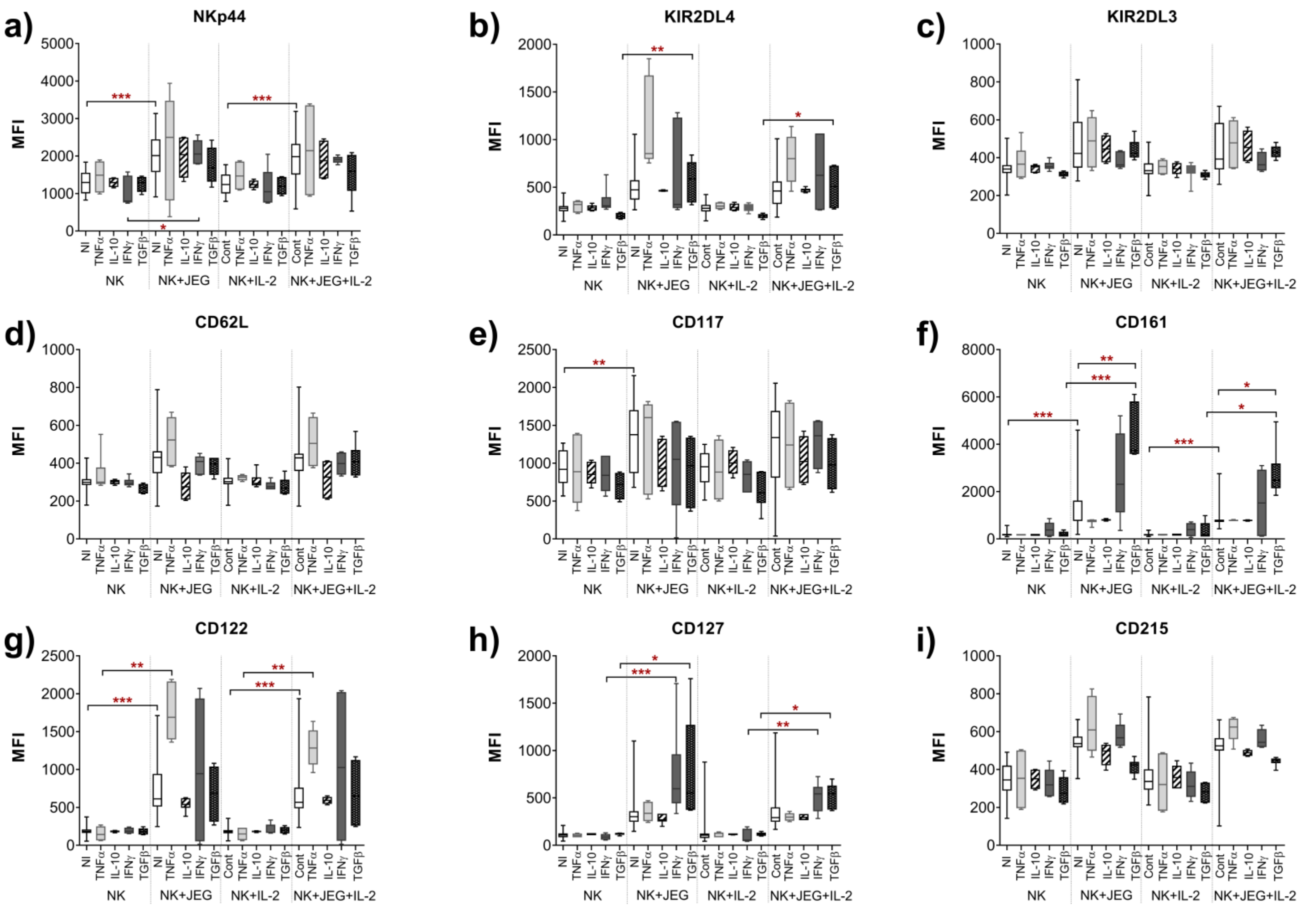

Supplementary Figure 2. The intensity of receptor **(a)** NKp44, **(b)** KIR2DL4, **(c)** KIR2DL3, **(d)** CD62L, **(e)** CD117, **(f)** CD161, **(g)** CD122, **(h)** CD127 and **(i)** CD215 expression by NK cells among the entire pool of NK-92 cells, after culturing in monoculture and coculture with JEG-3 cells, with cytokines TNFα, IL-10, IFNγ and TGFβ. NI (no inducer)—culturing without inducers. Cont (control)—culturing with IL-2. The experiments with each of the cytokines were conducted separately, twice, with three replicates in each experiment. Significant differences: *—*p* <0.05, **—*p* <0.01 and ***—*p*<0.001 (Kruskal–Wallis test).
